# Advice quality and source disclosure shape trust in AI-generated ethical advice

**DOI:** 10.1038/s41598-026-44258-1

**Published:** 2026-03-19

**Authors:** Lennart Meincke, Gideon Nave, Christian Terwiesch

**Affiliations:** 1 The Wharton School, University of Pennsylvania, Philadelphia, USA; 2 WHU–Otto Beisheim School of Management, Vallendar, Germany

**Keywords:** artificial intelligence, algorithm aversion, ethical decision-making, trust, advice utilization, Human behaviour, Psychology

## Abstract

**Abstract:**

Can AI deliver ethical advice of expert quality? Will humans trust it? We address these questions using dilemmas published in the New York Times (NYT) column, The Ethicist, comparing humans’ perception of AI-generated advice (GPT-4) to that of the NYT expert. First, we investigated how useful AI-advice is perceived by diverse participants (*N* = 187), including laypeople, MBA students, and an expert panel of scholars and clergy. We find that people perceive the quality of AI-generated ethical advice to be on par with that of expert advice, with no significant difference in usefulness ratings between the two sources. When given a direct choice, 57% of participants preferred AI-generated advice. Building on these findings, we conducted a three-condition experiment (*N* = 642) investigating how much humans are willing to accept this advice. Our results reveal that before observing the advice, humans display a strong algorithm aversion in this context, with 72.6% of participants preferring to be advised ethically by humans. After being exposed to the quality of the AI-generated advice, however, algorithm aversion is reduced substantially to 53.2%. When hiding the source of the advice, algorithm aversion is further reduced to 46.3%. Taken together, our findings suggest that while humans initially exhibit strong resistance to AI-generated ethical advice, this aversion significantly diminishes when they experience the quality of AI guidance firsthand, indicating that trust in algorithmic ethical reasoning may be more malleable than previously assumed and could evolve as people gain direct exposure to AI’s capabilities in moral decision-making.

**Protocol registration:**

The stage 1 protocol for this Registered Report was accepted in principle on 13/09/24. The protocol, as accepted by the journal, can be found at 10.17605/OSF.IO/6FPW7.

**Supplementary Information:**

The online version contains supplementary material available at 10.1038/s41598-026-44258-1.

## Introduction

In recent decades, artificial intelligence (AI) has made notable strides in emulating and, in several instances, surpassing human intelligence across diverse domains. Milestones in this development include IBM’s Deep Blue beating Grandmaster Gary Kasparov at chess in 1997, IBM’s Watson clinching victory in “Jeopardy” in 2011, DeepMind’s AlphaGo defeating Go champion Lee Sedol in 2016, and the proficiency of Large Language Models (LLMs) such as ChatGPT in a myriad of academic examinations by 2022^1^. In 2023, GPT-4 has even demonstrated an ability to generate creative ideas^2^ and to provide solid medical advice^3^.

With the advancement of AI’s capability comes a growing set of potential use cases for the technology. In some cases, AI allows automation without any human involvement. For example, video and motion sensors combined with AI empower fully autonomous vehicles. In other cases, AI augments human decision-making to combine the best of human judgment and the analytical power of AI. For example, AI supports radiologists looking at x-rays^4^, and LLMs help researchers synthesize large volumes of textual data^5^. Either way, the frontier separating tasks that need to be executed by humans from tasks that can reliably be executed by technology seems to be constantly shifting in favor of AI^6^. On one end of a hypothetical human-to-AI continuum are optimization tasks that rely on large datasets to make accurate predictions, such as finding the fastest route from one location to another. Few, if anyone, would argue that humans should conduct such tasks better. But which tasks are on the other end of this spectrum? Put differently, which tasks must be carried out by humans (in vivo) rather than by a machine (in silico)?

Decisions that involve making ethical and moral judgments, such as determining the fairness of sentencing in legal cases^7,8^, prioritizing patients for treatment in healthcare settings^9,10^, or deciding on the course of action in autonomous vehicle dilemmas^11,12^, are a prominent domain where the usefulness of algorithms is disputed. In healthcare, for example, algorithms prioritizing treatments based on data-driven criteria must navigate the moral complexities of deciding who receives scarce resources, like organ transplants^13^ or critical care during pandemics^14^. Likewise, autonomous vehicles programmed to minimize harm in unavoidable accidents face ethical challenges when they must ‘choose’ between negative outcomes, such as injuring passengers or pedestrians^15^. These examples underscore the ongoing debate about the role of algorithms in contexts requiring nuanced ethical and moral considerations.

Some pioneering AI scholars have been skeptical about AI’s ability to make moral and ethical decisions without human guidance, an issue referred to as the “alignment problem,”^16^ and indeed, there is no shortage of doomsday predictions that rogue AI systems might be an extinction threat to humanity^17^. Unsurprisingly, many ethics and philosophy scholars have also argued that moral judgment is beyond what a computer can do^18,19^. Such judgments, in their view, require exposure to subjective experiences, including feelings of joy and suffering. Moreover, one might argue that ethics is dynamic (it changes over time) and context-dependent (it requires a nuanced understanding of the emotions and expectations of the human actors involved in the situation), keeping it out of reach for machine-based intelligence.

Nonetheless, one could argue that providing ethical advice is yet another bastion to be taken by technology. For more than two decades, computer scientists have experimented with so-called artificial moral experts^20,21^, which have seen implementations in the field of healthcare^22^. Most of this early work was based on rule-based AI^16^ and predates the widespread adoption of LLMs starting in 2023. Ethical advice typically takes the form of language and thereby seems particularly suitable for AI in LLMs. Moreover, ethical dilemmas have been the subject of human deliberations for centuries, meaning there is a paper trail of millions of books and articles that can be used as input materials for training such LLMs.

Recently, researchers have begun to explore the moral judgment capabilities of LLMs by comparing them to human experts. Previous work reported that humans could not distinguish ethical advice generated by ChatGPT from that of multiple professional sources, including the New York Times (NYT) Ethicist, studied here, and perceived the AI-based advice more favorably^23^. Other research observed that humans perceive GPT-4’s moral reasoning as superior to undergraduate philosophy students across multiple dimensions, including intelligence and trustworthiness^24^. In that study, however, participants could distinguish advice generated by GPT-4 and humans— perhaps because the LLM was not explicitly instructed to mimic a human expert in its response style. Finally, it has also been found that humans perceive the ethical advice generated by GPT-4o as superior to advice generated by a representative sample of humans across dimensions, including trustworthiness and correctness^25^. These effects persisted even when compared to the advice of the NYT Ethicist (studied here).

Importantly, all studies above employed samples of lay people without knowledge and experience in ethical decision-making—who might miss nuanced ethical issues or subtle flaws in the reasoning of LLMs. Furthermore, participants in these studies were unaware of the advice source, limiting information on how humans might interact with AI-powered ethics chatbots in real life. Our first research aim is to lend further credibility to this emerging research stream by generalizing findings to participants with greater expertise in ethical decision-making: (1) MBA students in an elite university—future business leaders trained to navigate complex decisions with high-stakes and competing interests; and (2) ethical experts consisting of academics and clergy—who possess deep theoretical and applied knowledge of ethics across domains and often serve as consultants or advisors on the topic. Our pilot study demonstrates that MBA students and ethics experts perceive ethical advice generated by LLMs to be as valuable as that of a human expert.

But even if AI can provide ethical advice of expert-like quality, the question remains whether humans will accept such advice. A stream of literature in Psychology describes a phenomenon known as advice utilisation^26^ and, when the advice is generated by an algorithm, as algorithm aversion^27^. This research explores the conditions under which humans underutilize algorithmic capabilities, even when these capabilities match or surpass those of their human counterparts. Scholars identified various factors contributing to algorithm aversion, including the performance gap between the algorithm and humans, as well as characteristics of the decision-makers^28,29^. A key element influencing algorithm aversion is the decision-making context. Research has shown that humans generally trust algorithms for making objective data-driven decisions. Still, they are more hesitant when making subjective decisions, most notably in scenarios that have moral implications^12,23,30–32^.

Our second research aim is to investigate whether algorithm aversion diminishes once the high quality of the algorithm’s ethical advice is evident. Though the literature on algorithm aversion is extensive, most of it predates the widespread use of LLMs, and previous studies were either hypothetical (i.e., did not provide participants with actual ethical advice generated by an algorithm) or provided “black box” algorithmic recommendations whose underlying rationale is not fully understood by humans^12,30,31^. We argue that humans may trust LLM-based ethical advice more than they do other algorithmic recommendations for three main reasons. First, LLMs provide advice via natural language, which includes articulation of reasoning and argumentation. As such, LLMs provide a transparent basis for the advice, which has been shown to promote trust in some contexts^33,34^. Second, advice in natural language may make AI appear more human-like and increase humans’ perception of its trustworthiness^35^. Third, the advice of LLMs can be thoroughly inspected, and its quality can be assessed before deciding whether to use it. As our pilot data shows, humans perceive the quality of ethical advice provided by GPT-4 to be notably high.

To investigate how witnessing the high quality of LLM-based advice affects algorithm aversion in the ethical domain, we compared humans’ willingness to receive advice from an LLM *apriori* (that is, before seeing the advice; Condition A) to their willingness *ex-post* (that is, after witnessing the advice; Condition B). We hypothesized that algorithm aversion would significantly reduce once helpful AI-based ethical advice in natural language is evident.

Our final aim is to explore a third question whose answer is critical for the widespread adoption of ethical AI-based advice: how revealing (versus omitting) information about the algorithmic source affects humans’ readiness to accept the advice, even once its expert-like quality is apparent (Condition C). Given the previous findings described above^12,23,30–32^, we hypothesized that even though the high quality of AI’s advice is obvious, some algorithm aversion would remain. That is, disclosing the source of AI-based ethical advice would hurt humans’ tendency to rely on it.

Taken together, our pilot data and main study illuminate the effectiveness of AI-based ethical guidance and its potential public acceptance. Answering these questions is valuable for understanding algorithm aversion and trust in AI within ethically sensitive contexts. Furthermore, it offers practical insights for integrating AI into socially delicate and ethically complex areas, such as the judiciary, healthcare, and law enforcement.

**Fig.1:**
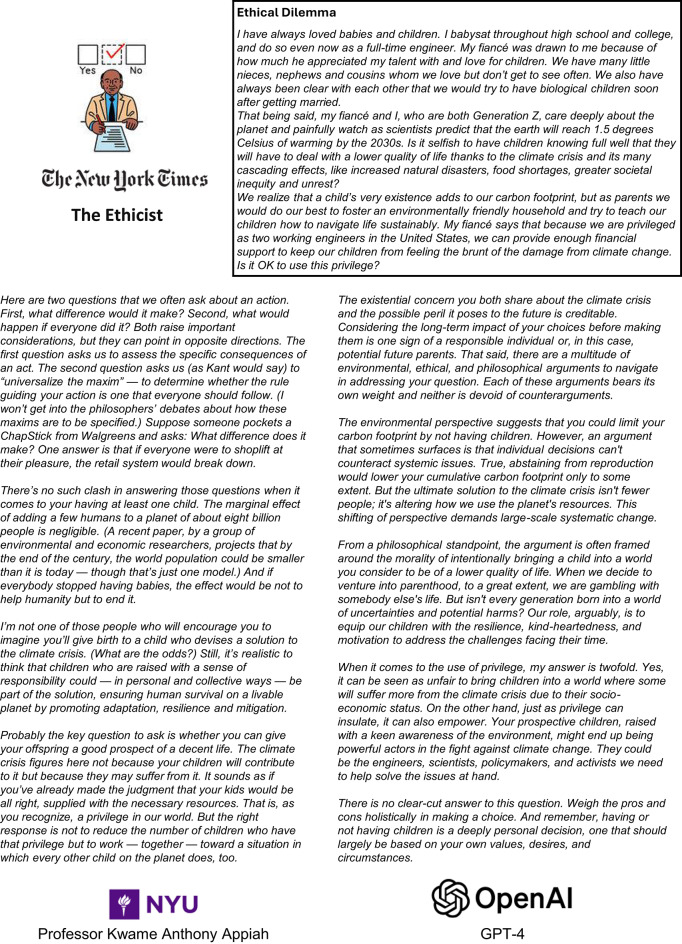
Task Illustration: An ethical dilemma that appeared in the NYT column The Ethicist (top), alongside advice generated by Professor Appiah (bottom left) and GPT-4 (bottom right).

## Study 1: Pilot

### Methods

#### Ethics Information

The Institutional Review Board (IRB) at the University of Pennsylvania approved the research described in this paper in September 2023, Protocol #854,365. All methods were performed in accordance with the relevant guidelines and regulations.

## Pilot data

We collected pilot data in September and October of 2023 (see ‘Data availability’ and ‘Code availability’ for additional information about materials, raw data, and analysis code). Our pilot study addressed our first research aim by generalizing previous findings that LLMs can provide high-quality, ethical advice with a single prompt^23–25^ to populations with knowledge and experience in ethical decision-making. To do this, we draw from an existing pool of twenty ethical dilemmas published in the NYT column “The Ethicist” alongside the analysis and advice by the expert Dr. Kwame Anthony Appiah—a Philosophy and Law professor at New York University (NYU), as used in previous research^23–25^. For each dilemma, we ask participants to compare the usefulness of Dr. Appiah’s advice to the advice generated by GPT-4 (see Fig. 1 for illustration). To ensure that stylistic differences in the language would not underlie the perceived usefulness of advice from the two sources^25^, we seeded GPT-4 with one example reply of Dr. Appiah’s writing and minimal prompting otherwise (see Supplementary Information 1). While results similar to our pilot study have been found without providing an example reply^23^, we believe we facilitate a clear comparison focusing on quality alone by keeping the style consistent. Importantly, all ethical dilemmas chosen were published in the NYT after the model release date of March 2023, overcoming an additional limitation of previous research. Previous work^25^ also uses data from The Ethicist, however, the knowledge cutoff of the chosen model (GPT-4o) at least partially overlaps with the sampled Ethicist questions and thus introduces the risk of potential bias. Our sample does not exhibit such bias.

The pilot study compared the perceived usefulness of the LLM and human expert’s ethical advice in three samples: (1) a panel of 18 experts in ethical decision-making, including four pastors, a rabbi, and 13 academics from well-known universities in the US and Europe. For participation, the experts received $250 as an Amazon gift card for themselves or a charitable organization. Out of the 18 experts, 17 provided responses (6 experts responded to the absolute condition and 11 to the relative one; we invited more experts for the relative condition since it was of greater interest to our study given the limited sample size overall; see further below); (2) a panel of 69 Wharton MBA students, who completed the task voluntarily outside of class; and (3) a panel of 100 participants on the Prolific platform that were paid $15 for the completion of our survey.

We randomized participants to two conditions. The first condition (“absolute”) showed participants a set of randomly chosen ethical dilemmas from the NYT pool alongside advice whose generating mechanism (human or AI) was chosen at random. Participants rated the perceived usefulness of the advice on a scale from 1 (not useful at all) to 7 (extremely useful). The second condition (“relative”) showed participants the dilemmas alongside human-generated and AI-generated advice in random order. We operationalize advice quality as the perceived usefulness in the absolute condition and the preference for advice in the relative condition. Participants had to choose whichever advice they found more useful. In both conditions, raters remained blind to the mechanism that generated the advice. Following the behavioral task, participants answered questions about their demographic background and previous familiarity with AI software and The Ethicist Column in the NYT (for summary statistics, see Supplementary Figure S1). Our analyses include the data of all participants but exclude responses provided less than 20 s after the introduction of the dilemmas (see distribution of response times in Supplementary Figure S2). After exclusion, the data includes 110 valid responses provided by experts (none excluded), 517 valid responses of prolific participants (16 excluded) and 263 valid responses of MBA students (46 excluded).

**Fig.2:**
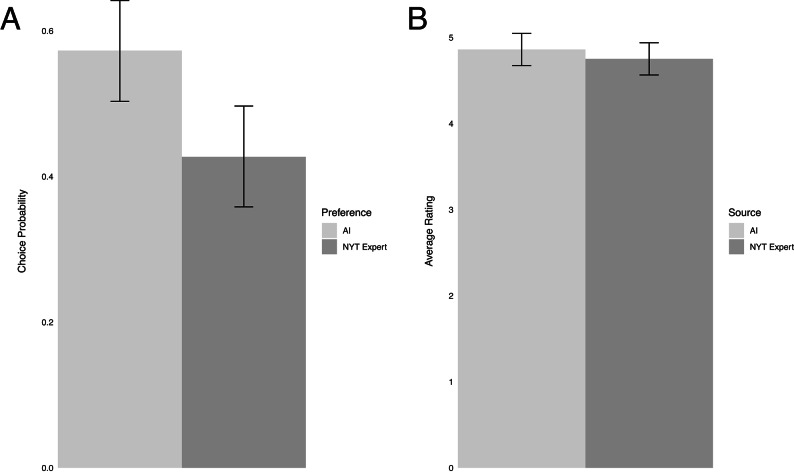
Pilot study results in the full sample. (**A**) Absolute condition. (**B**) Relative condition. Results are based on the regressions in Supplementary Tables S2 and S3.

Our pilot study established three foundational results (see Fig. 2 and Supplementary Table S1). First, the overall usefulness ratings of the ethical advice provided by both the human expert and AI provided were high. In the absolute condition, the average usefulness rating of our expert panel, consisting of academic experts and clergy, was slightly below five on a 7-point scale, with 37% of the advice receiving a rating of 6 or 7. Second, we find no evidence for a difference in perceived usefulness between the human expert and the AI in the absolute condition. To quantify this difference, we estimate a linear mixed model with the usefulness rating as the outcome variable and explanatory variables, including a fixed effect for the advice source (1 = AI; 0 = human) and random intercepts for participants and dilemma. The coefficient for advice source was small and insignificant, with 95% Confidence Intervals (CI) that excluded effects of |*d*| < 0.16 in favor of the human rater, which amount to 0.26 points out of a seven-point scale (*B* = 0.11; 95% CI [-0.26, 0.48]; *d* = 0.08; 95% CI [-0.16, 0.30]; *t*(80.6) = 0.58, *p* = .56; See Fig. 2 and Supplementary Table S2). The results were similar in regression models estimated for each of the three panels in isolation (see Supplementary Figure S3 and Supplementary Table S2).

Third, when given a choice (relative condition), we observed an increased preference for AI-generated advice in 57% of the choices of the whole sample. We quantified this effect by estimating the intercept of a mixed logistic regression model with random intercepts for participants and dilemmas in the whole sample (*B* = 0.29; 95% CI [0.01, 0.58]; OR = 1.34; 95% CI [1.01, 1.79]; *z* = 2.11, *p* < .035; See Fig. 2B and Supplementary Table S3). Using similar analyses in each of the three panels (see Supplementary Figure S4 and Supplementary Table S3), we found the most substantial effect among laypersons (Prolific sample), who favored the AI-generated advice 59.6% of the times (*B* = 0.43; 95% CI [0.14, 0.73 ]; OR = 1.54; 95% CI [1.15, 2.08]; *z* = 2.98, *p* < .003). The experts also opted for the AI advice more often (55.5%), though the effect was not statistically significant (*B* = 0.21; 95% CI [-0.67, 1.10 ]; OR = 1.23; 95% CI [0.51, 3.00]; *z* = 0.52, *p* < .604), likely due to lower statistical power resulting from the smaller sample. The MBA students also slightly preferred the AI advice (51.3%), though the effect did not reach statistical significance (*B* = 0.06; 95% CI [-0.29, 0.41]; OR = 1.06; 95% CI [0.95, 1.51]; *z* = 0.36, *p* < .717). As our additional research questions concern judgments of humans in general (i.e., not specific to experts), we focused on recruiting participants for our primary research study on the Prolific platform.

## Study 2: algorithm aversion

### Methods

#### Ethics information

The Institutional Review Board (IRB) at the University of Pennsylvania approved the research described in this paper in September 2023, Protocol #854,365.

## Main study

Having established that LLMs can provide high-quality ethical advice, we turn to the second question: will humans trust this advice? Specifically, do they exhibit algorithm aversion—discounting the guidance not because of its content or usefulness, but simply because it originates from an algorithm? Furthermore, we examine whether such aversion persists even after the high quality of the advice has been demonstrated.

**Fig.3:**
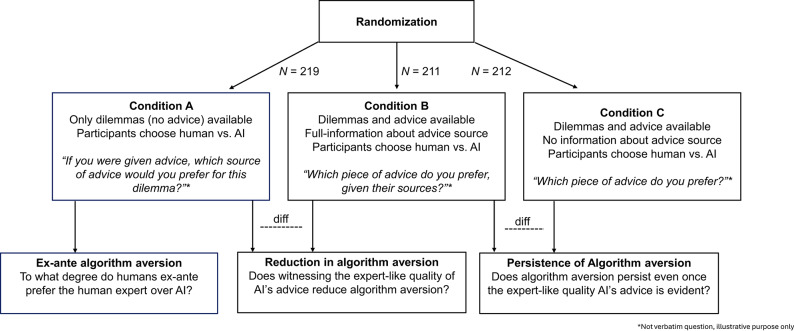
Experimental Design and Hypotheses

Our pre-registered study relied on a between-subject design that randomized *N* = 658 (slightly more than powered for since the survey did not close until all 618 required participants finished) participants to three experimental conditions of equal size (see Fig. 3; Table 1). Data collection and analysis was blind to the labels of the experimental conditions. According to our power simulations (see Supplementary Information 2), our study had more than 95% statistical power to detect the minimal treatment effect size of interest across the two studies, which amounts to a change of 5% in the probability of preferring the AI advice to the advice of a human expert, relative to a 60% baseline (observed in our pilot study).

In all experimental conditions, we showed participants the same ethical dilemmas published by the NYT as in our pilot study. Each participant saw five dilemmas randomly chosen from the original 20 dilemmas. After providing informed consent, participants answered a comprehension check (“Write in a single word how much 3 + 2 equals”; the registered protocol contained additional attention checks, see Protocol Deviations). We excluded questions for which participants fail to answer the comprehension question correctly. After applying exclusion criteria, our final sample consisted of 642 participants distributed across three randomised conditions (see Supplementary Table S4 for sample demographics).

In Condition A (“a priori”, *N* = 219), we showed participants only the dilemmas (i.e., without the advice) and asked them if they would rather receive advice from a human expert or an AI-based system in the form of ChatGPT (*“Put yourself into the shoes of the reader who submitted this question. Would you prefer to receive advice from a human expert or from an AI such as ChatGPT?”*). Based on previous research^12,30,31^, we hypothesized that humans will display algorithm aversion and significantly prefer to receive advice from a human expert.

Condition B (“full disclosure”, *N* = 211) showed participants the dilemmas alongside the actual advice generated by AI and the NYT expert in random order. We truthfully disclosed the advice source via text displayed above each piece of advice and ask participants to indicate their preferences (*“Put yourself into the shoes of the reader who submitted this question. In your opinion*,* which of the two pieces of advice is more useful to the reader?”*; the registered question was worded slightly differently, see Protocol Deviations*)*. By comparing the proportion of choices favoring the AI in Condition A to that in Condition B, we tested whether algorithm aversion diminishes once the expert-like quality of the AI’s advice is apparent.

Condition C (“no disclosure”, *N* = 212) is identical to condition B, except that participants were blind to the advice source. By contrasting the proportion of participants who favor the AI to the human expert in Condition C to that of Condition B, we tested whether algorithm aversion has remained even once the expert-like quality of AI’s ethical advice is observed.

Taken together, our pilot study and pre-registered experiment (i) established that human experts judge advice generated by an LLM as equally valuable as advice generated by a human expert, (ii) investigated whether observing the expert-like quality of AI-based advice mitigates algorithm aversion in the ethical domain, and (iii) evaluated the degree that algorithm aversion persists even after accounting for the high quality of AI-based ethical advice.

**Table 1:** Design Table

Question	Hypothesis	Sampling plan (e.g. power analysis)	Analysis Plan	Interpretation given to different outcomes
Does witnessing the expert-like quality of AI’s advice reduce algorithm aversion?	Greater preferences for receiving AI advice in Condition B relative to Condition A	*N* = 206 per condition Exclude responses made within less than 20 s of stimuli presentation, and participants who fail to correctly answer a comprehension question. After exclusions, we expect 97.2% statistical power to detect an effect size as small as beta = 0.21, at the 0.05 level. This effect corresponds to a change of 5% from a 60% baseline or a 2% from a 90% baseline.	Estimate a mixed model logistic regression on the pooled data of Conditions A and B, with the binary outcome variable representing choice (‘0’ = human, ‘1’ = AI), fixed effect for Condition (‘0’ = A, ‘1’ = B) and random intercepts for participants and dilemmas. We hypothesize a significant positive beta coefficient for Condition, quantified via a two-sided z-test.	Significant positive coefficient implies reduction in algorithm aversion once the advice quality is evident. Significant negative coefficient implies increase in algorithm aversion once the advice quality is evident. Insignificant coefficient implies no evidence for change in algorithm aversion once the advice quality is evident.
Does algorithm aversion persist, even once the high quality of AI’s advice is evident?	Greater preferences for receiving AI advice in Condition C relative to Condition B	*N* = 206 per condition Exclude responses made within less than 20 s of stimuli presentation, and participants who fail to correctly answer a comprehension question. After exclusions, we expect 97.2% statistical power to detect an effect size as small as beta = 0.21, at the 0.05 level. This effect corresponds to a change of 5% from a 60% baseline or a 2% from a 90% baseline.	Estimate a mixed model logistic regression on the pooled data of Conditions B and C, with the binary outcome variable representing choice (‘0’ = human, ‘1’ = AI), fixed effect for Condition (‘0’ = B, ‘1’ = C) and random intercepts for participants and dilemmas. We hypothesize a significant positive beta coefficient for Condition, quantified via a two-sided z-test.	Significant positive coefficient implies algorithm aversion persistence even once accounting for AI’s advice quality Significant negative coefficient implies that once accounting for AI’s advice quality, people prefer AI to a human expert Insignificant coefficient implies no evidence for algorithm aversion once the advice quality is evident.

## Analysis plan

Our first hypothesis was that participants will be more comfortable with AI’s ethical advice once they witness how good it is. In other words, exposure to the advice will significantly increase the proportion of decisions favoring the AI from Condition A to Condition B. We tested this hypothesis by estimating a mixed logistic regression model on Condition A and Condition B pooled data, with the binary outcome variable representing choice (‘0’ = human, ‘1’ = AI). The model included random intercepts for participants and dilemmas. The explanatory variable of interest was a fixed effect for Condition (‘0’ = Condition A, ‘1’ = Condition B). Our hypothesis corresponded to a positive beta coefficient for the Condition, and we formally tested it by calculating its statistical significance via a two-sided z-test.

Our second hypothesis was that even once the expert-like quality of AI’s advice is accounted for, algorithm aversion persists. That is, disclosing the advice source will reduce the proportion of decisions favoring AI in Condition B relative to Condition C. We tested this hypothesis by estimating a mixed logistic regression model on Condition B and Condition C pooled data, with a binary outcome variable representing choice (‘0’ = human, ‘1’ = AI). The model included random intercepts for participants and dilemmas. The explanatory variable of interest was the Condition (‘0’ = Condition B, ‘1’ = Condition C). Our hypothesis corresponded to a positive beta coefficient for the Condition, formally tested by calculating its statistical significance via a two-sided z-test.

Based on our simulations (Supplemental Information 2), our study had 97.4% statistical power to detect an effect size as small as beta = -0.21, corresponding to a change of 5% from a 60% baseline at the 0.05 (two-sided) level.

## Results

**Fig.4:**
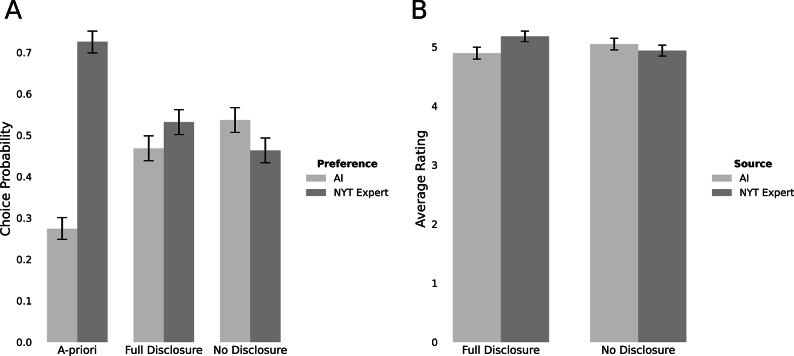
Main study results. (**A**) Advice preference. (**B**) Advice ratings. Results are based on the Regressions in Table 2 and Table 3. Note: *N* = 642. A: Each bar shows the observed proportion of AI-preferred responses in that condition. Error bars are 95 % CIs for the individual proportions; overlap (or lack thereof) should not be interpreted as a statistical test of differences between conditions. Formal comparisons are reported in the text and Table 2. B(exploratory): Each bar shows the average rating of each advice. Error bars are 95% CIs forthe average ratings and should not be interpreted as a statistical test of differences between conditions. Formal comparisons are reported in the text and Table 3.

Figure 4A displays the rates at which participants chose advice from different sources across the three experimental conditions and Table 2 summarizes the results of the corresponding analysis.

In the a-priori condition, participants preferred to rely on AI advice in only 27.4% of the dilemmas. However, in the full-disclosure condition, when participants were presented with high-quality advice of disclosed sources, these rates almost doubled (46.8%), and were significantly greater than in the a-priori condition (*B* = 0.96; 95% CI [0.72, 1.21]; OR = 2.62; 95% CI [2.05, 3.35]; *z* = 7.65, *p* < .001). Notably, in the no-disclosure condition, once the source information was hidden, the choice rate of the AI advice increased even further, to 53.7% (*B* = 0.32; 95% CI [0.10,0.55], OR = 1.38; 95% CI [1.10, 1.73]; *z* = 2.81, *p* = .005). These results support our two hypotheses, that (1) once the high-quality of AI becomes apparent, people are more likely to choose it, and (2) disclosing the source of the advice generates algorithm aversion even once the high quality is observed, though this aversion is less pronounced than in the a-priori condition.

**Table 2:** Summary of mixed logistic regression models predicting preference of AI-generated answers

Variable	A-priori v. Full Disclosure	Full Disclosure v. No Disclosure
Intercept	-1.109***	-0.154
Condition	0.962*** 95% CI [0.715, 1.209]	0.323** 95% CI [0.098,0.548]
**Random Effects (SD)**		
Participant (Intercept)	0.788	0.668
Dilemma (Intercept)	0.308	0.617
***N***	2150	2115
***N***_*Participant*_	430	423
***N***_Question_	20	20
DF Residual	2146	2111

Note. * *p* < .05, ** *p* < .01, *** *p* < .001. Positive condition coefficients indicate an increase in AI advice preference over human expert advice.

### Exploratory analyses

Across the conditions that included advice (B and C), advice from both sources was generally rated highly, around 5 in a seven-point scale (see Fig. 4B). However, preference for source was affected by disclosure conditions as quantified by an interaction between condition and information (*B* = -0.39; 95% CI [-0.57, -0.22]; *d* = -0.25; 95% CI [-0.36, -0.14]; *t*(3784.05) = -4.40, *p* < .001). Specifically, when the source was visible (Condition B), expert advice was rated higher than AI advice (*B* = 0.28; 95% CI [0.16, 0.41]; *d* = 0.18; 95% CI [0.10, 0.26]; *t*(3784.05) = 4.47, *p* < .001, see Table 3). When the source was not disclosed, the point estimate of this effect became negative and did not reach statistical significance (*B* = -0.11; 95% CI [-0.24, 0.01]; *d* = -0.07; 95% CI [-0.15, 0.01]; *t*(3785.66) = -1.74, *p* = .082, see Supplementary Table S6). These results indicate that when the source is disclosed, participants perceive the human expert advice to be more useful, but once the source is hidden AI is perceived on par with or even better than the expert.

**Table 3:** Summary of linear mixed model with the usefulness rating as the outcome variable. Explanatory variables included a fixed effect for the condition and advice source (1 = AI; 0 = human), and their interaction, plus random intercepts for participants and dilemmas.

Variable	Full Disclosure v. No Disclosure
Intercept (Condition B, Full Disclosure)	4.895***
Source (Expert = 1)	0.284***
Information (No Disclosure = 1)	0.152
Source x Information	-0.395***
***N***	4230
***N***_*Participant*_	423
***N***_Question_	20

Note. * *p* < .05, ** *p* < .01, *** *p* < .001. Positive coefficients indicate that the AI advice was rated higher than the expert advice. See Supplementary Table S5 for full specifications.

To further investigate the robustness of our preferences results across different stimuli, we use stimulus plots^36,37^(see Supplementary Figures S7 and S8). In the comparison of choices between the a-priori versus full-disclosure conditions (Supplementary Figure S7), we find that four dilemmas show an effect reversal that was inconsistent with a sampling error. In these dilemmas, participants preferred AI advice less after observing its quality (dilemmas 10, 1, 7 and 3; ranked in order of negative effect). A qualitative analysis suggests that these dilemmas are less emotionally loaded than the others (e.g., moving unattended beach gear or paying admission to a confederate historical site, as opposed to dealing with infidelity). Furthermore, the human expert provided clear directives on what to do in response to these dilemmas, whereas the AI’s advice was more abstract. We hope that future research will further explore this potential boundary condition for preferring human to AI advice. Finally, we did not find outlying stimuli when comparing responses between the full disclosure and the no disclosure condition.

## Discussion

Our results provide three key findings about the potential use of AI for ethical advice. First, our pilot study shows that a currently available AI model (GPT-4) is capable of providing ethical advice that is perceived as highly useful and of expert level quality. This result was replicated in our main study, as well as in research of others^23–25^. Our second, pre-registered study demonstrates that once the high quality of AI-based advice is evident, initial aversion to relying on AI for ethical advice is significantly reduced, with the fraction of dilemmas where AI was preferred increasing from 27% to 47%. Nonetheless, non-disclosure of the source further increases preferences for the AI-generated advice to 53.7%, indicating that some level of algorithm aversion still persisted.

Taken together, these findings suggest that the central question in the debate over AI in moral reasoning should shift from *“Can AI deliver ethical advice of expert quality?”* to *“Will humans trust it?”* While our results affirm the former, the answer to the latter is more nuanced. Once ChatGPT demonstrates high competence in producing ethical guidance, there is a significant increase in the share of people who are willing to take its advice even when its source is disclosed. However, people still discount the usefulness of the advice only because it was generated by AI (53% to 46%), displaying continued algorithm aversion.

While our findings do not attest to the mechanisms underlying the effect, we propose that apart from the high quality of the advice, the nature of LLMs introduces a distinct pathway for trust formation compared to more quantitative AI systems, such as those used in forecasting or classification tasks. Because LLMs communicate in natural language complete with reasoning, argumentation, and rhetorical nuance, their advice may appear more human and their thought processes more transparent. This linguistic familiarity may soften initial skepticism and accelerate the development of trust.

Our findings carry important implications for the deployment of AI in ethically charged settings, such as healthcare triage, legal decision support, or public policy consultation. While AI can produce high-quality ethical reasoning, premature disclosure of its machine origin might, to some degree, inhibit its adoption, particularly in early stages of use. This presents a practical dilemma: how to balance the potential benefits of AI-generated ethical advice with the normative expectations around transparency and informed consent. One possible strategy is to emphasize the substance and reasoning of AI advice over its provenance. Organizations may, for instance, present AI-generated recommendations alongside human expert opinions, allowing users to engage with the arguments before learning their source. Such an approach could facilitate trust formation while preserving users’ autonomy to assess the validity of the guidance. Still, this strategy must be approached cautiously, as it raises difficult ethical questions about disclosure, manipulation, and epistemic fairness.

Our study has several limitations that suggest directions for future research. First, we used a specific type of ethical dilemma (those published in The Ethicist) which may not generalize to all forms of moral decision-making. Future research should examine algorithm aversion across different types of ethical decisions and contexts. Additionally, as noted in our protocol deviations, the preference question in Conditions B and C asked which piece of advice was more useful rather than which source participants would prefer to follow, shifting emphasis from advice utilization to comparative content evaluation. The observed reduction in algorithm aversion from Condition A to Conditions B/C may thus also reflect a framing shift, rather than exposure to AI’s advice quality alone. The B-versus-C comparison, by contrast, remains a clean test of source disclosure on evaluations of identical advice. Separately, due to a programming error, the planned attention checks following each dilemma were not administered. Although we apply an initial comprehension check, reduced attention screening may have increased measurement noise (and thus potentially attenuated effects). Second, the participants in our main study were laypeople recruited from an online platform. While this provides ecological validity for general public attitudes, future research should examine how algorithm aversion varies among different professional groups who regularly make ethical decisions, such as the experts we enrolled in our pilot study.

Third, our study examined immediate expressions of preferences rather than actual decision-making behavior over time. Longitudinal research could examine whether algorithm aversion in ethical contexts decreases with repeated exposure to high-quality AI advice. Fourth, our AI advice was generated by GPT-4 with specific prompting to match human expert style. Future research should examine how different AI systems, prompting strategies, or presentation formats affect algorithm aversion in ethical contexts. Finally, our participants jointly evaluate the usefulness of the advice and how the advice was delivered in writing. Future research should disentangle the effect of the substantive quality of the advice provided by LLM from its ability to articulate it in a smooth and persuasive manner.

## Conclusion

To state the obvious, the ability of traditional formats—such as newspaper columns—to provide high-quality, personalized ethical advice at scale is fundamentally limited. AI systems like ChatGPT offer an alternative, with the potential to overcome these constraints and significantly enhance ethical decision-making across society. However, realizing this potential requires more than technical sophistication. Previous studies have shown that AI-generated advice is often regarded with notable resistance. Here, we show that once the high quality of the advice, which is provided transparently and in natural language, is apparent, people are less reluctant to adopt it—even in ethically charged domains. However, some aversion to reliance on AI still persists, suggesting that acceptance hinges not only on the content of the guidance but also on perceptions of its source.

The successful integration of AI into moral discourse will thus demand strategies that foster trust and legitimacy in algorithmic advice. These findings carry implications for AI governance, transparency, and public communication, particularly as AI systems begin to operate in domains historically reserved for human judgment. AI lacks consciousness, personal experience, and material interests. Yet, paradoxically, it can offer sound advice even in ethically charged domains. All models are, to some degree, simplifications, but this does not preclude them from offering meaningful ethical support. As we move forward, the challenge will be not just to improve the advice AI can give, but to create the social conditions under which we are willing to hear it.

## Electronic Supplementary Material

Below is the link to the electronic supplementary material.

Supplementary Material 1

## Data Availability

Raw data and materials are available at https://osf.io/pz2sf/.

## Declarations

### Protocol Deviations

Our Stage 1 registered protocol specified a target sample size of 618 participants based on an a priori power analysis. However, our final sample (prior to applying exclusion criteria) included 658 participants. This discrepancy occurred because the survey remained open slightly beyond the target, and additional responses were collected before the system automatically closed data collection.

Additionally, we identified an unintentional deviation in the wording of the preference question used in Conditions B and C. While the pre-registered version stated: *“Put yourself into the shoes of the reader who submitted this question. In your opinion*,* which of the two sources of advice would you prefer to follow?”*, the version implemented in the study was: *“Put yourself into the shoes of the reader who submitted this question. In your opinion*,* which of the pieces of advice is more useful to the reader?”* This change was the result of a human error in survey programming.

Finally, our pre-registered design included additional attention checks following each dilemma. Due to a technical error, only the initial attention check was presented to participants in the deployed survey. No subsequent attention checks appeared as originally planned.

We transparently report these deviations and believe they do not compromise the integrity or interpretability of the pre-registered analyses.
