# Supplementary material for: Advice quality and source disclosure shape trust in AI-generated ethical advice
Source: Sci Rep. 2026 Mar 19;16:11868. doi: 10.1038/s41598-026-44258-1 (PMC13065772; doi:10.1038/s41598-026-44258-1)
Supplement: Supplementary file 1 — Supplementary Material 1 [file 41598_2026_44258_MOESM1_ESM.docx]

# **Supplementary information**

1. System Prompt
2. Power Simulations
3. Supplemental Figures
4. Supplemental Tables
5. **System Prompt**

Our system prompt described the model’s task, provided it with one example reply from Dr. Appiah, and instructed it to write in a similar style. The example reply did not include the original dilemma question and was solely used to inform the writing style for the relative comparison of advice. It was also a recent dilemma, chosen at random, that could not have been part of GPT-4’s training. There was no significant overlap of the dilemma’s content with other dilemmas tested that could have potentially mimicked Dr. Appiah’s advice. Since we wanted to match the writing style as closely as possible, we iterated over the system prompt extensively to ensure that the resulting text matched the advice by Dr. Appiah closely in length and paragraph format.

An analysis of Dr. Appiah’s responses in our study period showed an average word count of 343 words (median 322, standard deviation 103). We prompted GPT-4 to also aim for this same format. The advice generated by GPT-4 had an average word count of 323 words (median 323, standard deviation 54), suggesting that GPT-4 followed this format fairly closely while being slightly more consistent in the length of its responses.

Since LLMs are not designed to count words, we experimented with different prompts that include the number of paragraphs and sentences. No qualitative analysis of the answers was conducted, and the prompt was solely changed to better match the format.

| **System Prompt**  You are an ethicist answering reader questions. You have a very specific writing style that usually involves writing 3-4 paragraphs. You also use first person narration. Example:  *What your mother and her husband are planning to do, as it happens, is at odds with much rabbinical thought concerning inheritance. A Judaic scholar I conferred with confirms that the mainstream Talmudic tradition of Halakha, or Jewish law, revered by the Orthodox Union, holds that apostates don’t forfeit their right to inherit. (Maimonides would allow a rabbinical court to fine an apostate at its discretion — but the permission is not given to the parents.) And then marrying a non-Jew isn’t as severe a transgression as apostasy; so if an actual apostate retains the right to inherit, it’s clear that someone who has merely married a non-Jew does as well. You might think that it’s awkward to penalize your kids for departing from Halakha by departing from Halakha yourself. But picking and choosing from the traditions you are going to respect is a widespread practice among Jews and gentiles alike.*  *The real question is whether the scheme is wise or decent. I fear that it is neither. That your siblings now have an incentive to postpone marriage until your parents are dead raises doubts about its wisdom. That your siblings might marry someone acceptable to the Orthodox rabbinate in order to secure this inheritance raises doubts about its decency. Whom we marry is properly up to us. Parents may express their views; coercion, though, is wrong. Does threatening to deprive someone of a substantial inheritance amount to coercion? Different understandings of coercion will come out differently on this. But it’s too close for comfort.*  *You suggest that once your mother and stepfather decided not to leave money to a child who hadn’t married the right kind of Jew, it would have been better had they kept it to themselves. That’s an odd conclusion, but a cogent one: They should have restricted themselves to morally acceptable forms of suasion. In the meantime, you might encourage them to discuss their codicil with a rabbi, who could explain to them what the Jewish sages had to say on the subject.*  Each paragraph should be long and contain 6-8 sentences. On occasion, you use much shorter or longer paragraphs. Rarely, you might make a reference to the "readers" of your answers, but most of the time you do not. |
| --- |

1. **Power Simulations**

Our main study investigated two research questions corresponding to two hypotheses (see Table 1). We expected the smallest effect-size for the second hypothesis, comparing the proportion of participants favoring AI’s advice to the human expert between Condition C and Condition B. We thus performed power calculations for the minimal effect size of interest in this test (5% from the 60% baseline observed in the Prolific panel in our pilot study), assuming random assignment with equal probability to each treatment group.

We performed power analysis using the ‘lme4’ and ‘simr’ libraries in R, designed for mixed-effects models and power simulation, respectively. We generated data using a logistic regression model, where responses are binary outcomes denoting choice (‘0’ = human, ‘1’ = AI). The simulation performed 1,000 iterations for each sample size to assess the stability of the power estimate across different random data generations. We simulated new datasets with varying sample sizes in increments of 50 participants (see Figure S5). This involved generating participants and dilemmas effects drawn from normal distributions with variances determined by the Intraclass Correlation Coefficient (ICC) and randomly assigning each participant to one of two treatment conditions with 50% probability. Akin to our main study, each participant responds to questions about a subset of 5 out of 20 dilemmas, ensuring that questions cannot be repeated per participant.

Our simulation modeled the probability of a positive response using the logistic function incorporating an intercept, the treatment effect (i.e., Condition B vs. Condition C), and the effects of participants and dilemma. We set the intercept parameter of the logistic model to 𝛽_0_ *= 0.412*, which corresponds to preferences for the AI advice 60% of the time, based on our pilot data from the prolific panel (similar to Condition C). We set the fixed treatment effect to 𝛽_1_ = *-0.205*, which corresponds to the minimal effect size of interest: 5% reduction in preferences for AI, from the 60% baseline (Condition C) to 55% (in Condition B). (This effect also corresponds to a 2% preference reduction from a 90% baseline). Based on our pilot study, we set the ICC to *0.193*, indicating the proportion of the total variance in responses explained by differences between participants.

After generating the data, we fit a mixed-effects logistic regression model (R function ‘glmer’), with the binary response as the outcome and explanatory variables that included a fixed treatment effect (for condition) and random intercepts for participants and dilemmas as predictors.

We simulated responses for a varying number of participants to detect the appropriate sample size for obtaining a statistical power of 95% or more. This involved generating many datasets and testing whether the treatment effect is statistically significant (*p* < 0.05, two-sided) in each simulated dataset. The power is the proportion of simulations where the treatment effect is statistically significant.

The output of our simulation included the estimated power for detecting the treatment effect, its associated 95% confidence intervals, and the estimated effect size. We repeated this process for each sample size to get a distribution of power estimates, providing a comprehensive view of the power of the study under the specified model and parameters.

Figure S8 displays the results, which indicate that a sample of *N* = 175 per condition (*N* = 525 for the full sample, which includes three experimental conditions), provides 97.2% statistical power (CI [96%, 98.1%]) to detect a reduction in the proportion of choices favoring the AI from 60% (Condition C) to 55% (Condition B). We set our final sample to *N* = 618 to account for a 15% exclusion rate.

1. **Supplemental Figures**

**Supplementary Figure S1. Pilot Samples Characteristic Distributions.** Summary of participants’ responses to questions about their demographic background and previous familiarity with AI software and The Ethicist Column in the NYT


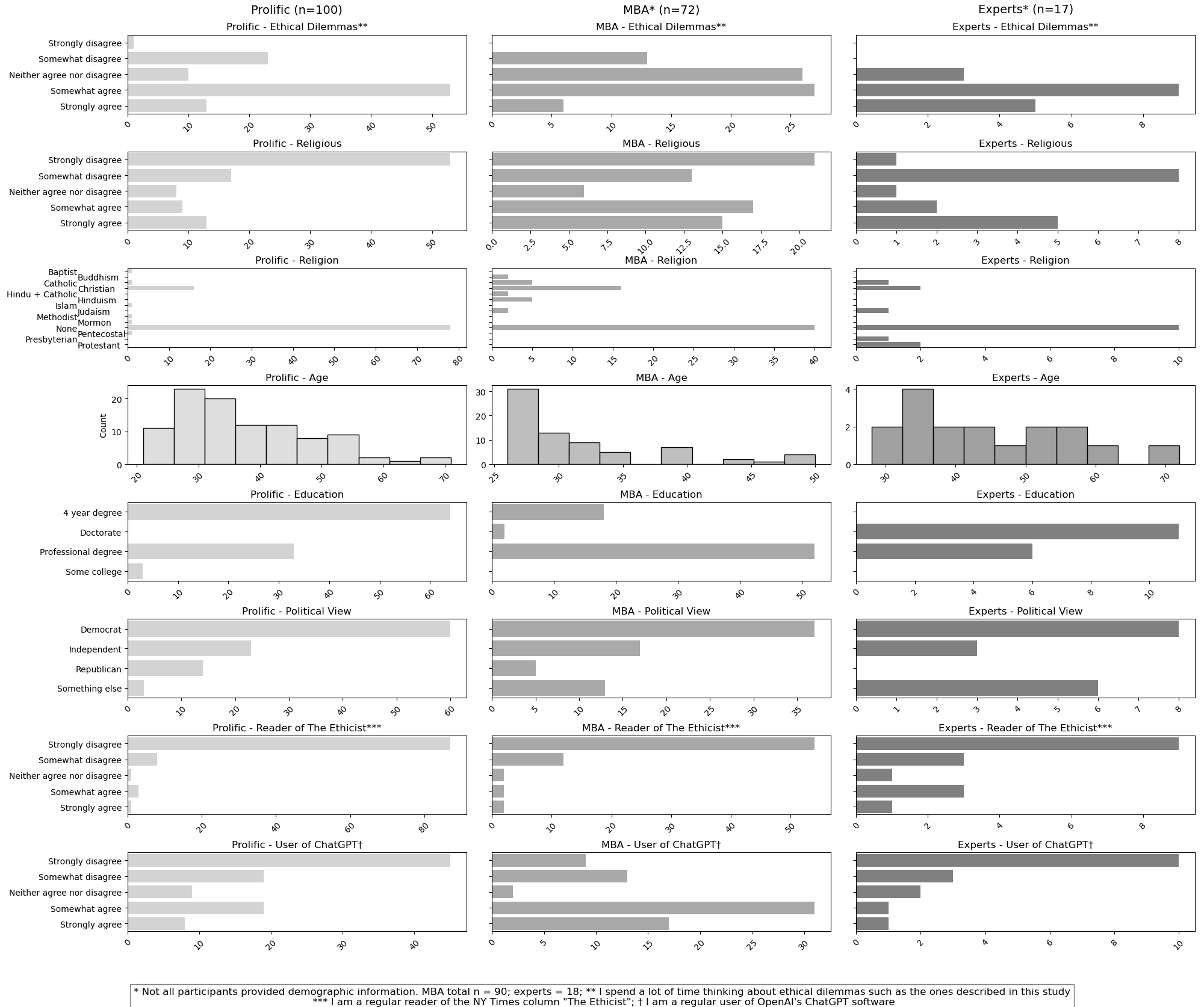


**Supplementary Figure S2. Pilot distribution of response times (in seconds) for each question item per panel.**


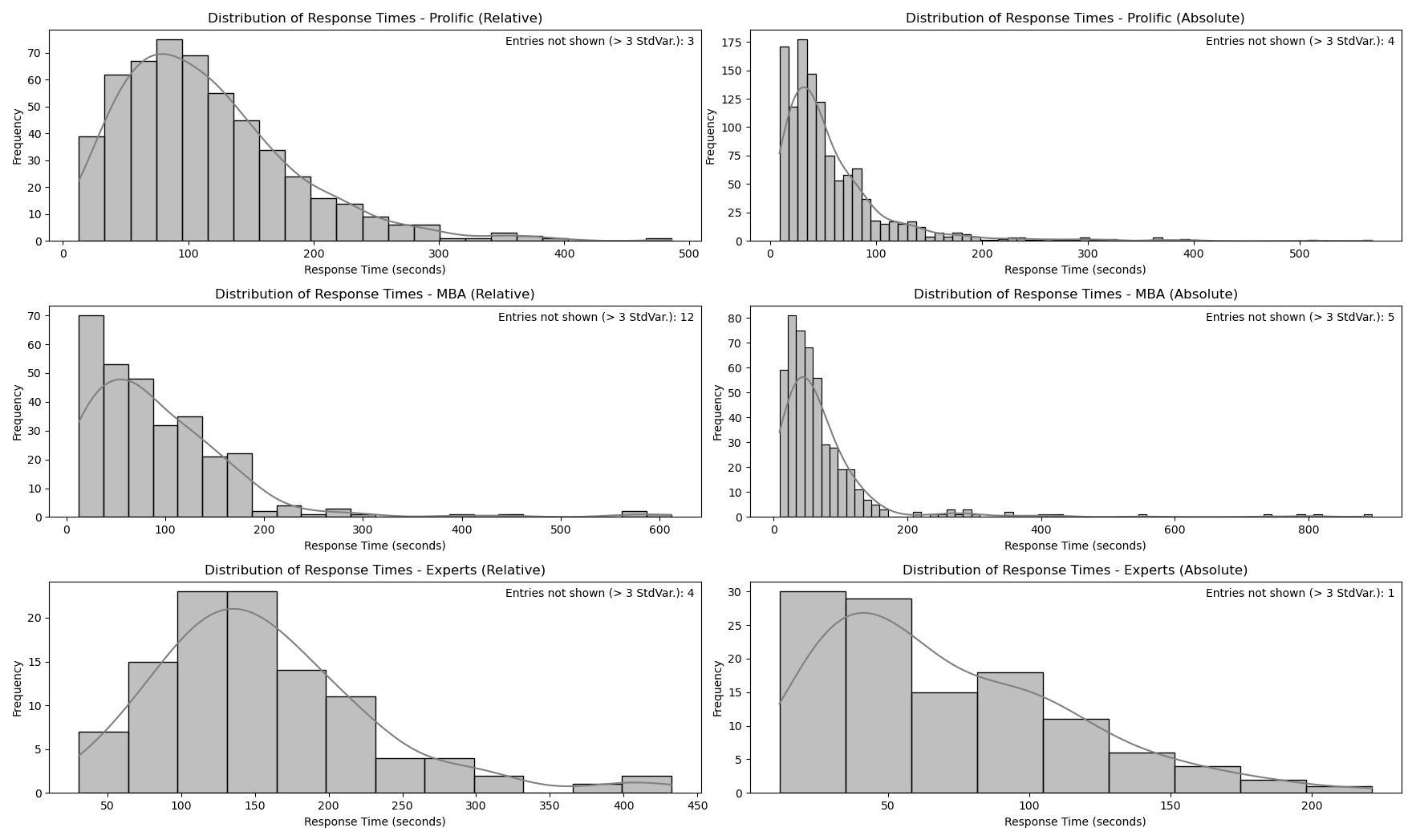


**Supplementary Figure S3. Pilot study, absolute condition results across panels.** Results are based on the regression in Table S2.

**
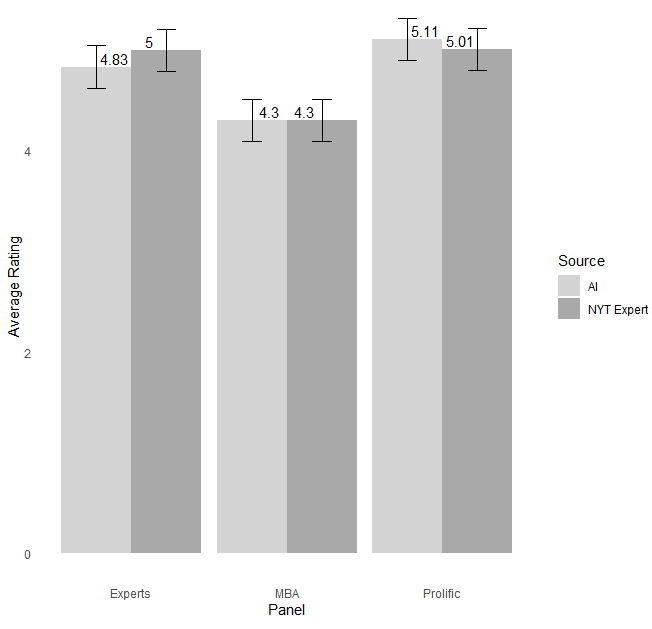
**

**Supplementary Figure S4. Pilot study, relative condition results across panels.** Results are based on the regression in Table S3.

**
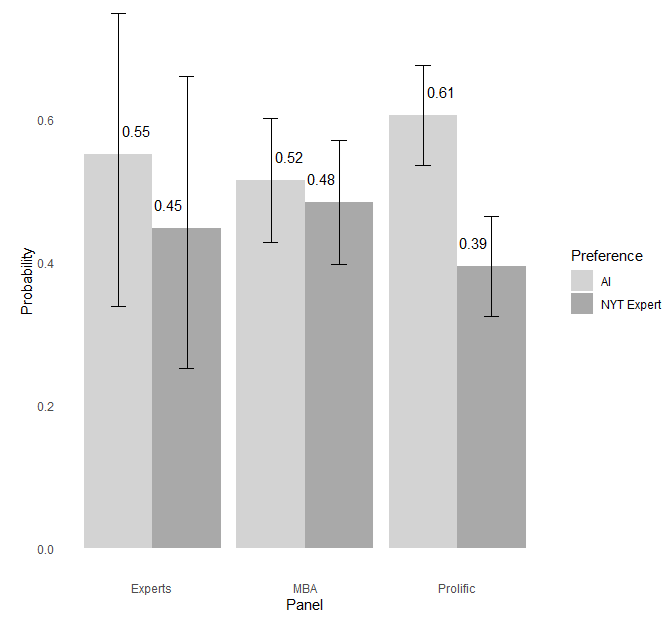
**

**Supplementary Figure S5. Power Simulations.** Logistic regression power simulation for detection of a treatment effect of *B* *= -0.21,* which corresponds to a change of 5% from a 60% baseline, at the 0.05 (two-sided) significance level.

**
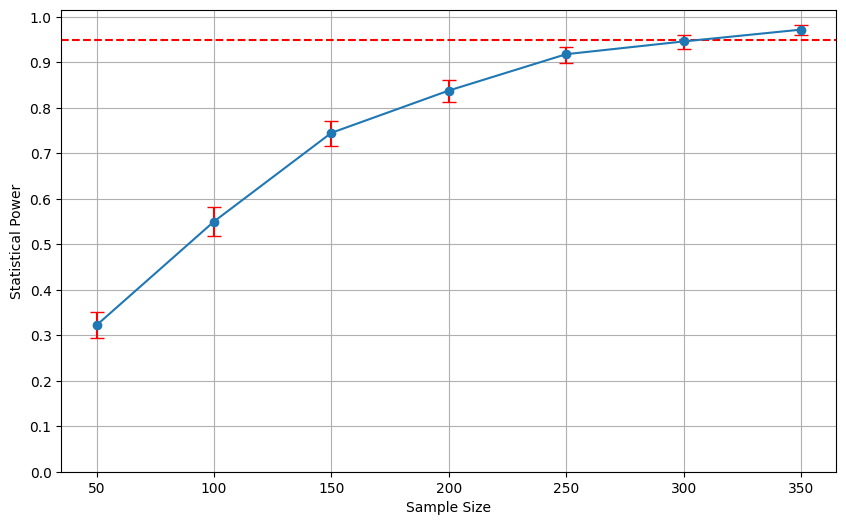
**

**Supplementary Figure S6. Main study distribution of response times (in seconds) for each question item per panel.**

**
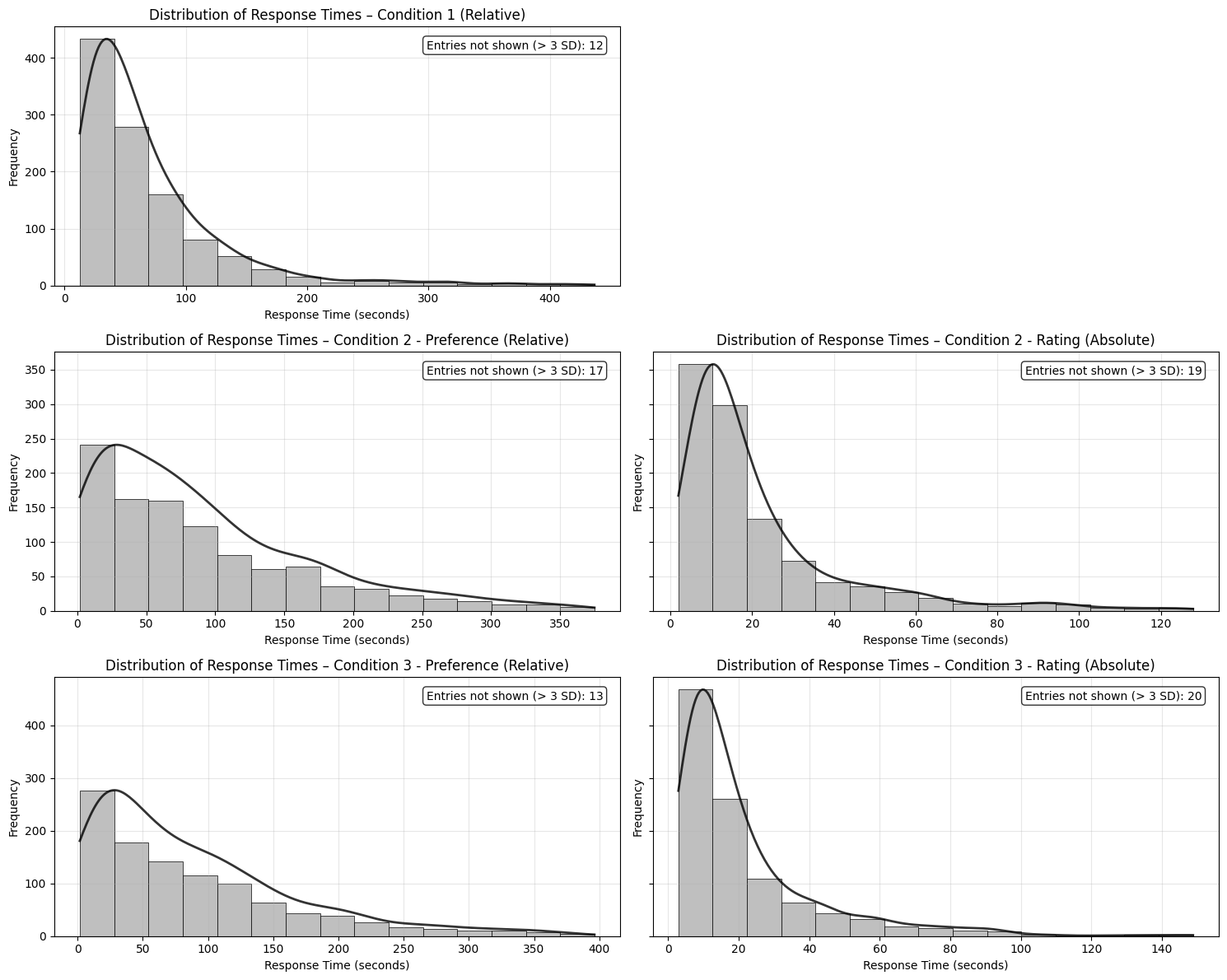
**

**Supplementary Figure S7. Main study, stimulus plot for a-priori versus full disclosure.
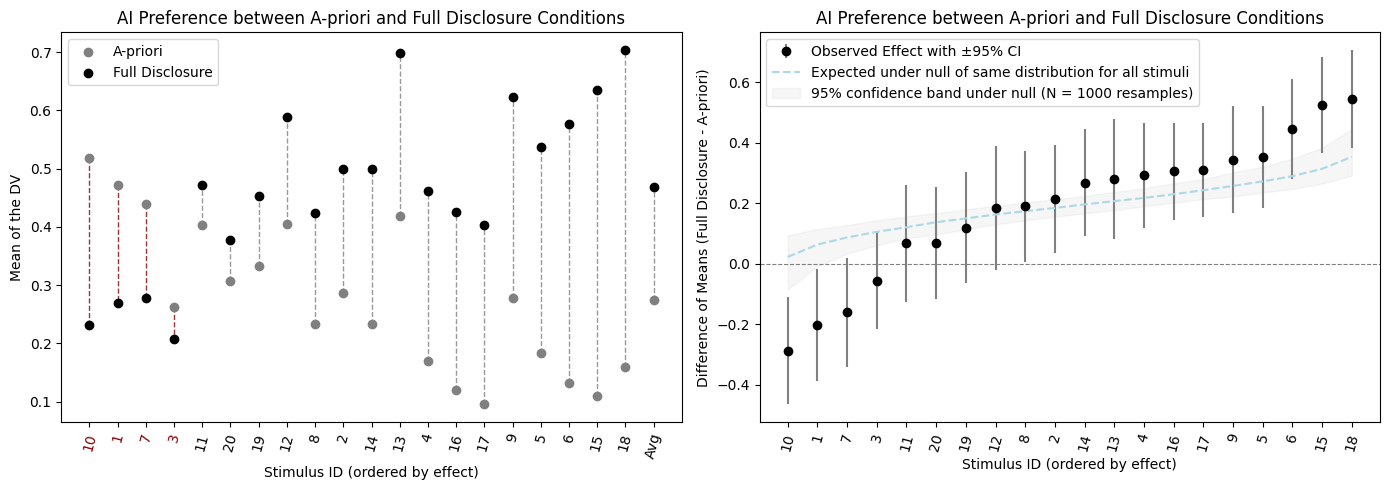
**

**Supplementary Figure S8. Main study, stimulus plot for full disclosure versus no disclosure.**

**
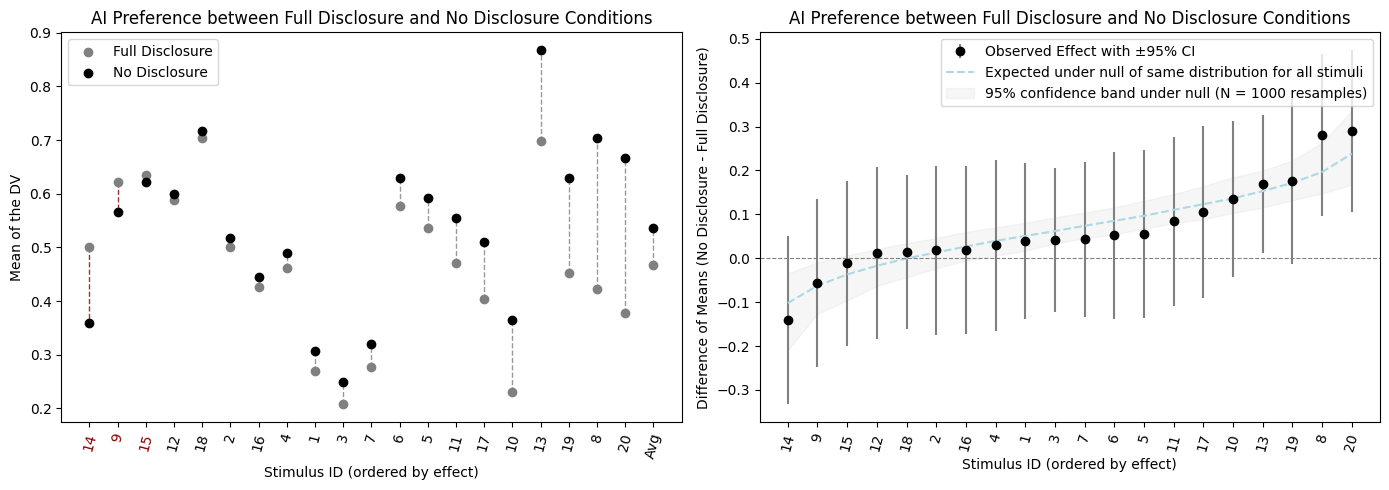
**

1. **Supplemental Tables**

**Supplementary Table S1. Pilot study, summary statistics.**

|  | **Subjects on Prolific**  **(Human \| AI)** | | **MBA students**  **(Human \| AI)** | | **Experts**  **(Human \| AI)** | |
| --- | --- | --- | --- | --- | --- | --- |
| **Absolute rating condition** | Avg: 5.03  Median: 5.0  Stdev: 1.50 | Avg: 5.1  Median: 5.0  Stdev: 1.50 | Avg: 4.27  Median: 5.0  Stdev: 1.67 | Avg: 4.21  Median: 5.0  Stdev: 1.71 | Avg: 4.94  Median: 5.0  Stdev: 1.34 | Avg: 4.92  Median: 5.0  Stdev: 1.37 |
| **Relative rating condition** | Preferred:  200 times  (40.40%) | Preferred:  295 times  (59.60%) | Preferred:  128 times  (48.67%) | Preferred:  135 times  (51.33%) | Preferred:  49 times  (44.55%) | Preferred:  61 times  (55.45%) |

**Supplementary Table S2. Pilot study, absolute condition results.** Linear mixed model with the usefulness rating as the outcome variable. Explanatory variables included a fixed effect for the advice source (1 = AI; 0 = human) and random intercepts for participants and dilemmas.

|  | **All Groups** | | | **Prolific** | | | **MBA** | | | **Experts** | | |
| --- | --- | --- | --- | --- | --- | --- | --- | --- | --- | --- | --- | --- |
| *Predictors* | *𝛽s* | *CI* | *p* | *𝛽s* | *CI* | *p* | *𝛽s* | *CI* | *p* | *𝛽s* | *CI* | *p* |
| (Intercept) | 4.75 | 4.44 – 5.06 | **<0.001** | 5.01 | 4.65 – 5.37 | **<0.001** | 4.30 | 3.83 – 4.77 | **<0.001** | 5.00 | 4.44 – 5.56 | **<0.001** |
| Source  [AI = 1] | 0.11 | -0.26 – 0.48 | 0.560 | 0.10 | -0.34 – 0.54 | 0.660 | 0.00 | -0.63 – 0.63 | 0.997 | -0.17 | -0.95 – 0.62 | 0.673 |
| **Random Effects** | | | | | | | | | | | | |
| σ^2^ | 1.87 | | | 1.68 | | | 2.20 | | | 1.87 | | |
| τ_00_ | 0.51 _Participant_ID_ | | | 0.43 _Participant_ID_ | | | 0.39 _Participant_ID_ | | | 0.00 _Dilemma_ | | |
|  | 0.16 _Dilemma_ | | | 0.16 _Dilemma_ | | | 0.29 _Dilemma_ | | | 0.04 _Participant_ID_ | | |
| ICC | 0.26 | | | 0.26 | | | 0.24 | | |  | | |
| N | 84 _Participant_ID_ | | | 50 _Participant_ID_ | | | 28 _Participant_ID_ | | | 6 _Participant_ID_ | | |
|  | 20 _Dilemma_ | | | 20 _Dilemma_ | | | 20 _Dilemma_ | | | 20 _Dilemma_ | | |
| Observations | 750 | | | 460 | | | 230 | | | 60 | | |
| Marginal R^2^ / Conditional R^2^ | 0.001 / 0.264 | | | 0.001 / 0.261 | | | 0.000 / 0.238 | | | 0.004 / NA | | |

**Supplementary Table S3. Pilot study, relative condition results.** Logistic mixed model with the choice (1 = AI; 0 = Human) as the outcome variable. Explanatory variables included random intercepts for participants and dilemmas.The intercept quantifies the difference from 50% choice probability in log odds.

|  | **All Groups** | | | **Prolific** | | | **MBA** | | | **Experts** | | |
| --- | --- | --- | --- | --- | --- | --- | --- | --- | --- | --- | --- | --- |
| *Predictors* | *OR* | *CI* | *p* | *OR* | *CI* | *p* | *OR* | *CI* | *p* | *OR* | *CI* | *p* |
| (Intercept) | 1.34 | 1.02 – 1.76 | **0.035** | 1.54 | 1.16 – 2.04 | **0.003** | 1.06 | 0.77 – 1.48 | 0.716 | 1.23 | 0.56 – 2.68 | **0.603** |
| **Random Effects** | | | | | | | | | | | | |
| σ^2^ | 3.29 | | | 3.29 | | | 3.29 | | | 3.29 | | |
| τ_00_ | 0.33 _Participant_ | | | 0.34 _Participant_ | | | 0.09 _Participant_ | | | 0.95 _Dilemma_ | | |
|  | 0.21 _Dilemma_ | | | 0.09 _Dilemma_ | | | 0.18 _Dilemma_ | | | 0.63 _Participant_ | | |
| ICC | 0.14 | | | 0.12 | | | 0.07 | | | 0.33 | | |
| N | 102 _Participant_ | | | 50 _Participant_ | | | 41 _Participant_ | | | 11 _Participant_ | | |
|  | 20 _Dilemma_ | | | 20 _Dilemma_ | | | 20 _Dilemma_ | | | 20 _Dilemma_ | | |
| Observations | 868 | | | 495 | | | 263 | | | 110 | | |
| Marginal R^2^ / Conditional R^2^ | .000 / 0.141 | | | 0.000 / 0.116 | | | 0.000 / 0.075 | | | 0.000 / 0.325 | | |

**Supplementary Table S4. Main Study Samples Characteristic Distributions.** Summary of participants’ responses to questions about their demographic background and previous familiarity with AI software and The Ethicist Column in the NYT

| **Variable** | **Category / Statistic** | **n** | **%** |
| --- | --- | --- | --- |
| **Age (years)** | *Mean ± SD* | 39.97 ± 13.69 | — |
|  | *Median* | 36 | — |
| **Gender** | Female | 333 | 51.9 |
|  | Male | 309 | 48.1 |
| **Education** | 4-year degree | 307 | 47.8 |
|  | Professional degree | 181 | 28.2 |
|  | Doctorate | 56 | 8.7 |
|  | 2-year degree | 51 | 7.9 |
|  | Some college | 40 | 6.2 |
|  | Other | 7 | 1.1 |
| **Religion (affiliation)** | Christian | 274 | 42.7 |
|  | Other | 239 | 37.2 |
|  | Catholic | 93 | 14.5 |
|  | Protestant | 17 | 2.6 |
|  | Islam | 13 | 2.0 |
|  | Muslim | 6 | 0.9 |
| **Religious commitment** | Strongly agree | 251 | 39.1 |
|  | Somewhat agree | 214 | 33.3 |
|  | Strongly disagree | 127 | 19.8 |
|  | Neither agree nor disagree | 27 | 4.2 |
|  | Somewhat disagree | 23 | 3.6 |
| **Political view** | Republican | 309 | 48.1 |
|  | Democrat | 223 | 34.7 |
|  | Independent | 106 | 16.5 |
|  | Something else | 4 | 0.6 |
| **Ethical Dilemmas*** | Somewhat agree | 309 | 48.1 |
|  | Strongly agree | 149 | 23.2 |
|  | Neither agree nor disagree | 86 | 13.4 |
|  | Somewhat disagree | 86 | 13.4 |
|  | Strongly disagree | 12 | 1.9 |
| **Reads *The Ethicist***** | Strongly disagree | 226 | 35.2 |
|  | Somewhat agree | 178 | 27.7 |
|  | Somewhat disagree | 103 | 16.0 |
|  | Neither agree nor disagree | 89 | 13.9 |
|  | Strongly agree | 46 | 7.2 |
| **ChatGPT user***** | Somewhat agree | 271 | 42.2 |
|  | Strongly agree | 234 | 36.4 |
|  | Somewhat disagree | 62 | 9.7 |
|  | Neither agree nor disagree | 38 | 5.9 |
|  | Strongly disagree | 34 | 5.3 |
|  | Other | 3 | 0.5 |

*Note*. *I spend a lot of time thinking about ethical dilemmas such as the ones described in this study. **I am a regular reader of the NY Times column "The Ethicist". ***I am a regular user of OpenAI's ChatGPT software.

**Supplementary Table S5. Main study, absolute condition results.** Linear mixed model with the usefulness rating as the outcome variable. Explanatory variables included a fixed effect for the condition and advice source (1 = AI; 0 = human), and their interaction, plus random intercepts for participants and dilemmas.

|  | **Quality Rating (1-7)** | | |
| --- | --- | --- | --- |
| *Predictors* | *Estimates* | *CI* | *p* |
| (Intercept) | 4.895 | 4.742 – 5.049 | < .001 |
| Source (Expert = 1) | 0.284 | 0.160 – 0.409 | < .001 |
| Information (No Disclosure = 1) | 0.152 | -0.013 – 0.317 | 0.072 |
| Source x Information | -0.395 | -0.571 – -0.219 | < .001 |
| **Random Effects** | | | |
| σ^2^ | 2.131 | | |
| τ_00_ _Participant_ | 0.325 | | |
| τ_00_ _Question ID_ | 0.051 | | |
| ICC | 0.150 | | |
| N _Question ID_ | 20 | | |
| N _Participant_ | 423 | | |
| Observations | 4230 | | |
| Marginal R^2^ / Conditional R^2^ | 0.005 / 0.154 | | |

**Supplementary Table S6. Main study, absolute condition results.** Linear mixed model with the usefulness rating as the outcome variable. Explanatory variables included a fixed effect for the condition and advice source (1 = AI; 0 = human), and their interaction, plus random intercepts for participants and dilemmas. Uses Condition C as the baseline compared to table S5.

|  | **Quality Rating (1-7)** | | |
| --- | --- | --- | --- |
| *Predictors* | *Estimates* | *CI* | *p* |
| (Intercept) | 5.047 | 4.894 – 5.200 | < .001 |
| Source (Expert = 1) | -0.110 | -0.235 – 0.014 | 0.082 |
| Information (No Disclosure = 1) | -0.152 | -0.317 – 0.013 | 0.072 |
| Source x Information | 0.395 | 0.219 – 0.571 | < .001 |
| **Random Effects** | | | |
| σ^2^ | 2.131 | | |
| τ_00_ _Participant_ | 0.325 | | |
| τ_00_ _Question ID_ | 0.051 | | |
| ICC | 0.150 | | |
| N _Question ID_ | 20 | | |
| N _Participant_ | 423 | | |
| Observations | 4230 | | |
| Marginal R^2^ / Conditional R^2^ | 0.005 / 0.154 | | |
